# What to Look Out for in a Newborn with Multiple Papulonodular Skin Lesions at Birth

**DOI:** 10.3390/dermatopathology8030043

**Published:** 2021-08-17

**Authors:** Sylvie Fraitag, Olivia Boccara

**Affiliations:** 1 Department of Pathology, Necker-Enfants Malades Hospital, APHP, 75015 Paris, France; 2 Department of Dermatology, Necker-Enfants Malades Hospital, APHP, 75015 Paris, France; olivia.boccara@aphp.fr

**Keywords:** newborn, blueberry muffin rash, dermal erythropoiesis, leukemia cutis, metastatic neuroblastoma, metastatic rhabdomyosarcoma, metastatic rhabdoid tumor, Langerhans cell histiocytosis, multiple juvenile xanthogranuloma, infantile myofibromatosis, subcutaneous fat necrosis of the newborn, mastocytosis, multiple neonatal hemangiomatosis, lymphangioendotheliomatosis with thrombocytopenia, blue rubber bleb nevus syndrome, glomuveinous malformation

## Abstract

Multiple papulonodular skin lesions at birth can indicate the presence of various benign and malignant disorders. Although the lesions’ clinical aspect (color and consistency, in particular) may steer the clinician towards one disorder or another (infantile myofibromatosis, xanthogranuloma, or metastatic neuroblastoma), the diagnosis can only be confirmed by the histopathologic assessment of a biopsy. In neonates, a rapid but accurate diagnosis is critical because skin lesions may be the first manifestation of a malignant disorder like leukemia cutis or metastatic neuroblastoma. Here, we review the various disorders that may manifest themselves as multiple skin lesions at birth.

## 1. Introduction

Skin lesions that develop at birth or within the first weeks of life (i.e., during the neonatal period) may variously be papular, nodular, ulcerated, or crusted. The lesions color (flesh-colored, pinkish, reddish, brownish, or rather blue or violaceous), aspect (nodular, angiomatous, or that of a white peripheral halo), consistency (hard or soft), and the presence of associated systemic symptoms may steer the clinician towards one diagnosis rather than another. However, the clinical findings alone are usually insufficient for a firm diagnosis, and a skin biopsy is usually necessary. Skin lesions at birth have several possible causes, and it is important to bear in mind that malignant conditions can appear at this early age. Indeed, skin metastases may be the revealing signs of malignant neoplasms in neonates [1]. Hence, when multiple skin lesions are observed, the physician must do everything possible to deliver an accurate diagnosis as soon as possible and rule out malignancy if necessary. The skin biopsy should be as large and deep as possible and should be cut into two pieces, one of which should be frozen in case cytogenetic tests are subsequently needed.

## 2. Blueberry Muffin Rash

**Figure 1:**
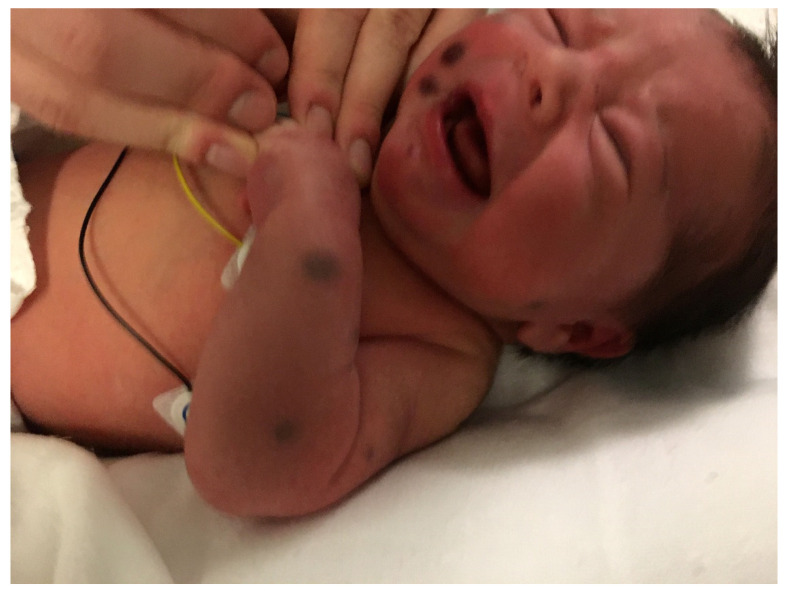
Blueberry muffin rash.

The “blueberry muffin baby” was first described in congenital rubella with thrombocytopenia [2]. It is characterized by bluish-red macules, papules, or nodules of dermal erythropoiesis primarily caused by intra-uterine infections with rubella virus, cytomegalovirus, or *Toxoplasma*. This rash can also be a sign of other serious systemic disorders, including congenital leukemia (Figure 1) (Table 1) [3,4]. A skin biopsy is mandatory for rapid diagnosis of the underlying disease.

**Table 1:** Causes of blueberry muffin rash.

**Figure 2:**
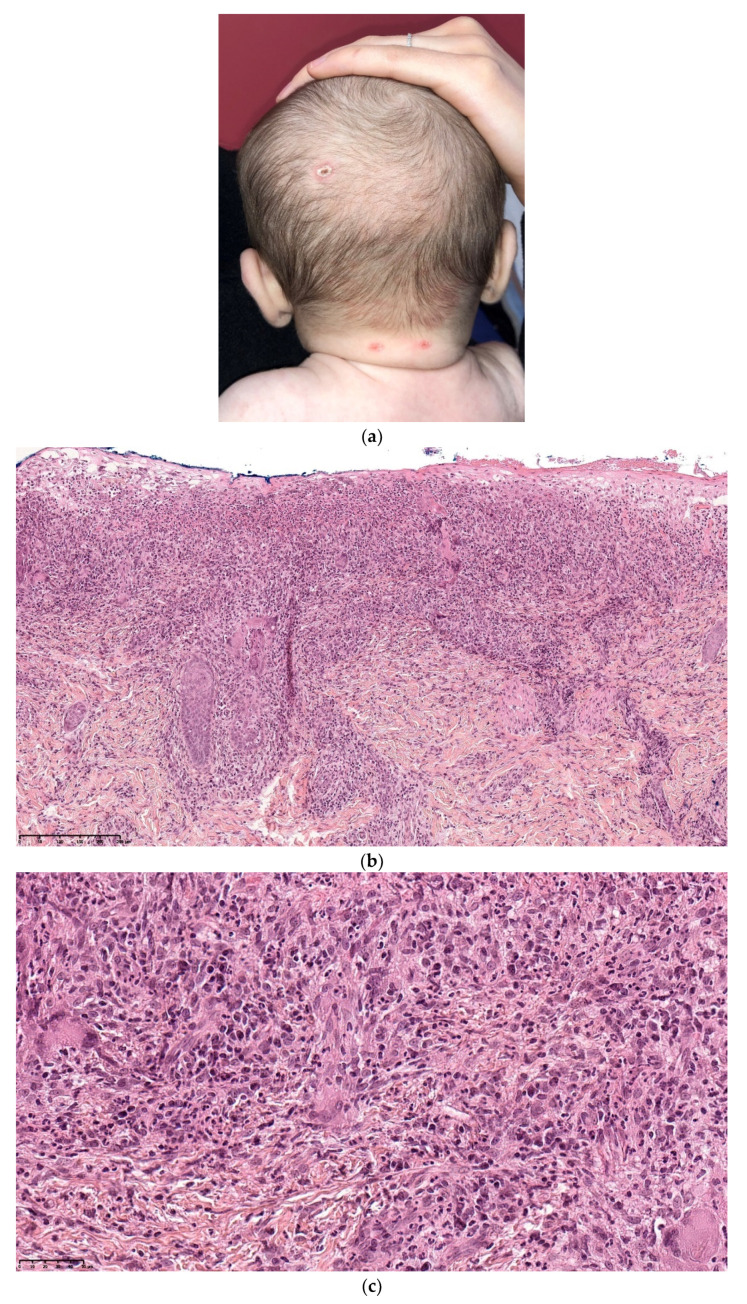

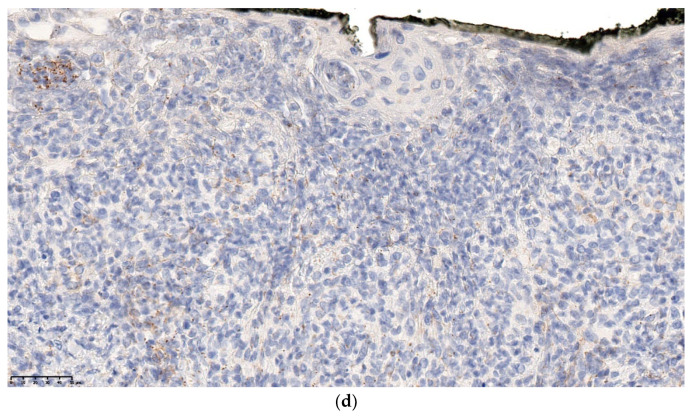
Congenital syphilis. (**a**) Multiple small erythematous lesions (by courtesy S Mallet). (**b**) 4× superficial and deep cellular infiltrate with ulcerated overlying epidermis (by courtesy N Macagno). (**c**) 25× polymorphic infiltrate containing plasma cells. (**d**) Positivity of anti-treponema showing numerous spirochetes between cells.

**Dermal Erythropoiesis**
• Congenital infections
Toxoplasmosis
Rubella
Cytomegalovirus
Herpes simplex virus
Coxsackie B2 virus
Syphilis (Figure 2a–d)
• Hemolytic disease of the newborn
Rhesus and ABO incompatibility
Hereditary spherocytosis
Twin-twin transfusion syndrome
**Neoplastic Diseases**
• Congenital leukemia
• Metastatic neuroblastoma
• Transitory myeloproliferative disease
• Langerhans cell histiocytosis
• Congenital metastatic rhabdomyosarcoma
• Metastatic rhabdoid tumor
• Disseminated juvenile xanthogranuloma
• Multicentric infantile myofibromatosis

### 2.1. Dermal Erythropoiesis

**Figure 3:**
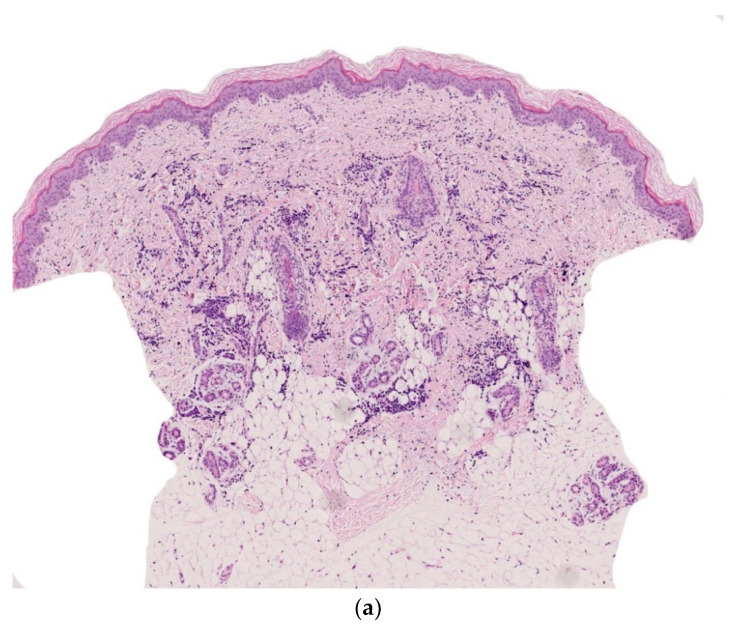

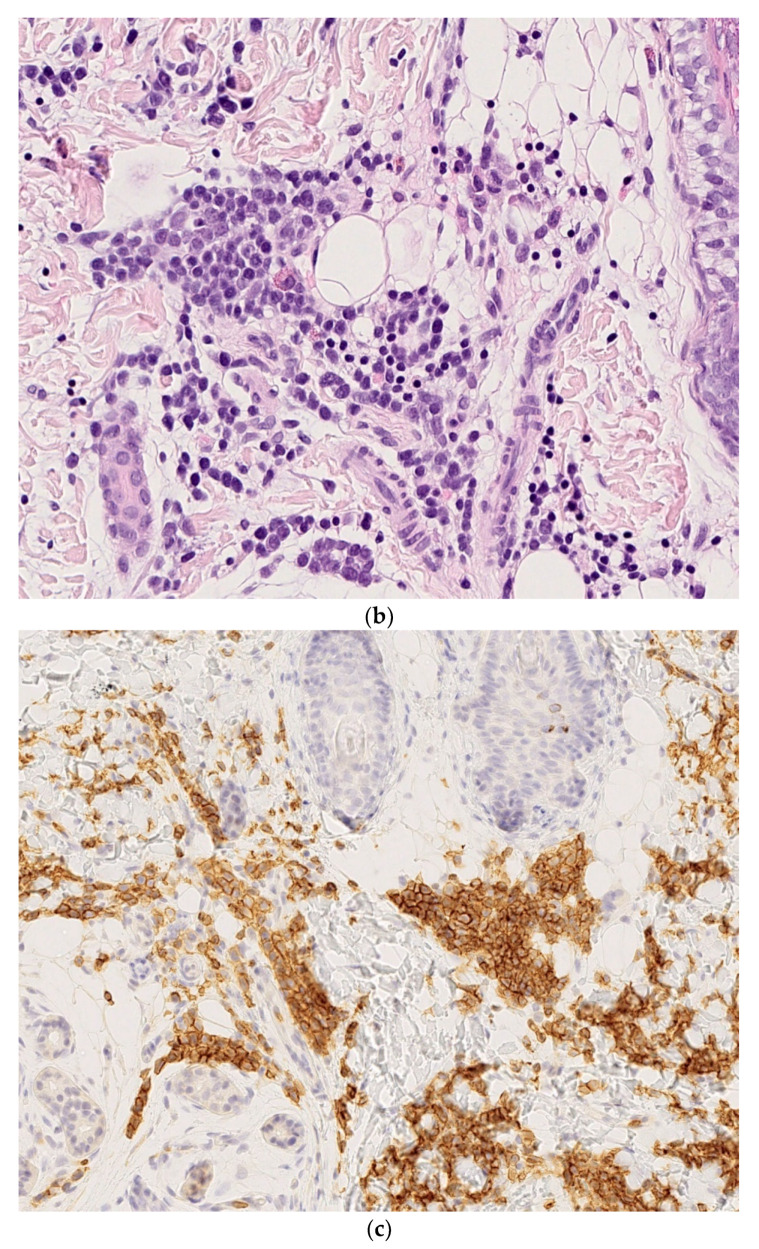
Dermal erythropoiesis (**a**,**b**) 2.5× and 25× clusters of erythroblasts in the dermis. They are highlighted by anti-glycophorin antibody (**c**).

Dermal erythropoiesis is easy to recognize histologically. It is characterized by the presence of clusters of erythroblasts in the superficial dermis and mid-dermis (Figure 3a–c). The erythroblasts are atypical and, to avoid confusion with leukemic blasts, can be highlighted by immunohistochemical staining with an anti-glycophorin antibody.

### 2.2. Neoplastic Diseases

#### 2.2.1. Leukemia Cutis

**Figure 4:**
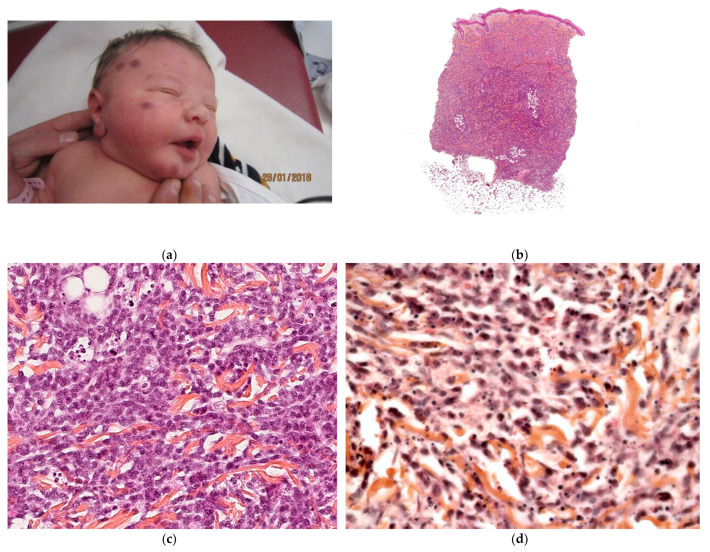

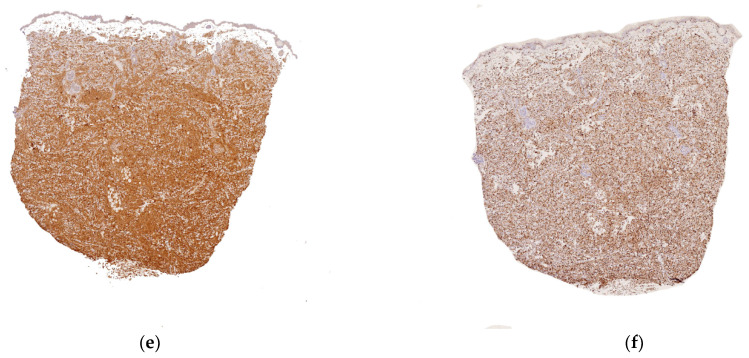
Congenital leukemia cutis: (**a**) blueberry muffin rash; (**b**) 4× dense dermal infiltration separated from the epidermis by a grenz-zone; (**c**) 10× medium-sized blastic cells arranged in single-file between collagen bundles and with a peri-vascular and peri-adnexal arrangement as well; (**d**) 25× apoptotic cells and mitotic figures; (**e**) CD68 intense positivity; (**f**) Ki67 immunostaining: almost 100% of nuclei are positive.

Acute leukemia is the most common malignancy that presents with specific skin lesions [1]. Most neonates have a high leukocyte count and hepatosplenomegaly. However, aleukemic leukemia cutis (characterized by leukemic cells that invade the skin alone before spreading to the bloodstream) is not rare. Although the skin lesions occur early, the blood cell count and even the bone marrow profile are normal for a while. The lesions may have a nodular, indurated, and bluish appearance (Figure 4a). Early diagnosis and treatment are critical.

##### Histology

In most cases, the diagnosis is straightforward. A dense dermal infiltrate is separated from the epidermis by a “grenz zone” and extends into the subcutaneous tissue with a peri-vascular and peri-appendageal arrangement. Cells are arranged in single file between the collagen bundles. They are medium-sized, with a high nuclear-cytoplasmic ratio, nuclear debris (apoptosis), and mitotic figures (Figure 4b–h). A good clue is the very high proportion of tumor cells, and almost no reactive inflammatory cells, in the infiltrate. CD68, lysozyme, and myeloperoxidase are the most sensitive immunohistochemical markers for detecting monoblasts (acute monocytic leukemia 5) or myelomonoblasts (acute monocytic leukemia 4), which account for the vast majority of leukemias at this age. In most cases, 90 to 100% of the cells are positive for KI67. Importantly, these immature monoblasts may lack CD163.

##### Outcome

Infantile leukemia and leukemia cutis can be very severe, lethal conditions but can also resolve spontaneously [5]. These disorders appear to be strongly linked to cytogenetic anomalies. For example, the 11q23 translocation appears to be linked to aggressive acute leukemia. In contrast, t(8;16) tends to be associated with spontaneous regression.

#### 2.2.2. Metastatic Neuroblastoma

**Figure 5:**
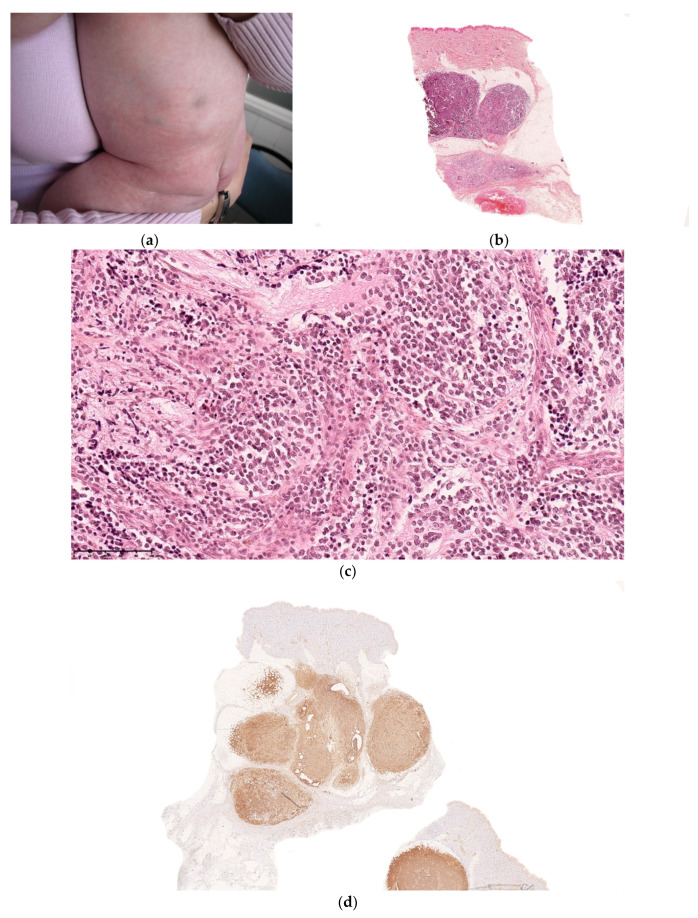
Metastatic neuroblastoma. (**a**) Pink lesion surrounded by a whitish halo. (**b**) 2.5× tumoral nodules in the reticular dermis and the subcutis. (**c**) 25× blue round cells. (**d**) Anti-synaptophysin immunostaining.

Neuroblastoma is the most common tumor of childhood and accounts for 32% of all neonatal tumors (Figure 5a). This tumor is unique because of its distinctive biological behavior and the large number of clinical manifestations. Multiple bluish cutaneous nodules (producing the ‘‘blueberry muffin rash’’) occur in more than a third of patients under 12 months of age and may constitute the initial sign of metastatic neuroblastoma [1]. A vaso-constrictive white halo around the nodules is suggestive of the latter disorder and should prompt the clinician to perform a biopsy without delay. Most infants with this sign are classified as stage IV-S.

##### Histology

Examination of a cutaneous nodule reveals a uniform, small-cell, malignant tumor and, in some cases, Homer–Wright pseudorosette formations. The tumor cells are positive for Phox2b and synaptophysin (Figure 5b–d). 

##### Outcome

Despite the presence of tumors in the liver, skin, and bone marrow, neonates with stage IV-S disease paradoxically have a relatively good prognosis. The tumor may remit or transform spontaneously into a benign ganglioneuroma.

#### 2.2.3. Transitory Myeloproliferative Disease

Transient myeloproliferative disorder (TMD) is a Down’s syndrome-specific spontaneously regressing neoplasm that affects up to 10% of neonates with the syndrome. In 20–30% of cases, TMD reoccurs as progressive acute megakaryoblastic leukemia (AMKL) at 2–4 years of age. The TMD and AMKL blasts are morphologically and immunophenotypically identical, and have the same acquired mutations in the *GATA1* gene [6].

##### Metastatic Rhabdomyosarcoma

Rhabdomyosarcoma (RMS) is a highly malignant neoplasm that accounts for most of the soft tissue sarcomas in neonates; it is the second most common after fibrosarcoma [7,8]. Girls outnumber boys by 3.3 to 1. The most frequent disease site is the limb, followed by the neck, the orbit of the eye, and the trunk. RMS accounts for 6% of the malignancies that metastasize into the skin. Although rare, cutaneous metastasis of RMS may be the revealing sign. The cutaneous metastases commonly form bluish cutaneous nodules [9].

##### Histology

Alveolar rhabdomyosarcoma is the main histological type involving the skin. The tumor comprises small- to medium-sized darkly staining cells with round nuclei and scant cytoplasm arranged in an alveolar pattern. The most diagnostically useful immunohistochemical markers are myogenin and MyoD1, followed by desmin. The diagnosis must be confirmed in a cytogenetic test.

##### Outcome

In one study, rhabdomyosarcoma had the second lowest survival rate (2 out of 13, 15%) after rhabdoid tumor (4%) [1]. The prognosis has not improved in recent years, despite changes in therapy [10].

##### Metastatic Rhabdoid Tumor

Metastatic rhabdoid tumor (MRT) is a highly malignant exceedingly rare neoplasm characterized clinically by rapid growth, early metastases, and a high mortality rate among neonates and infants [7]. Rhabdoid tumors rank fourth in order of incidence after leukemia, Langerhans cell histiocytosis, and neuroblastoma. MRT may present in the skin (especially in the head and neck area) as a solitary primary tumor or as one or more metastatic skin nodules [7]. Metastatic disease is present in more than half of fetuses and neonates at the time of diagnosis. The main skin sites for metastases are the limbs, trunk, face, and neck; several sites can be affected simultaneously. Almost all patients present with blue cutaneous nodules.

##### Histology

**Figure 6:**
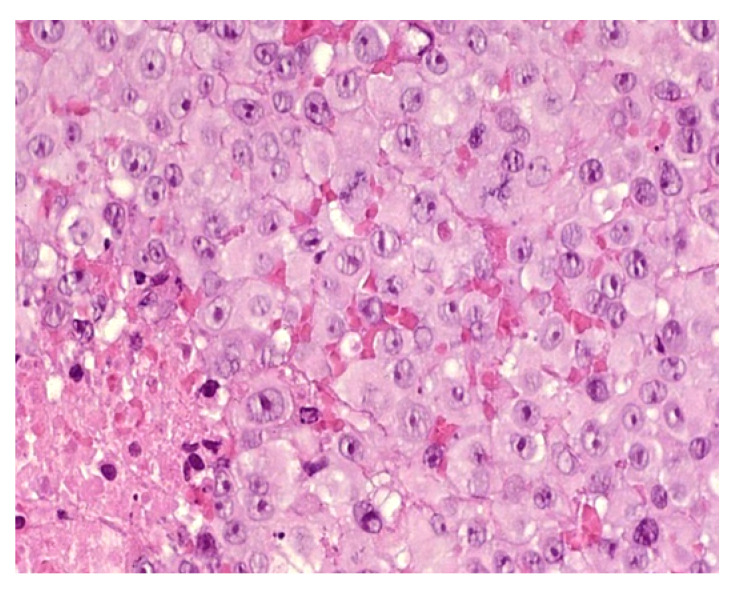
40× Rhaboid tumor. Very atypical large cells.

Classically, MRT is described as sheets of polygonal cells with abundant acidophilic cytoplasm, eccentric round vesicular nuclei, prominent nucleoli, and periodic acid Schiff-positive hyaline cytoplasmic inclusions (Figure 6). Loss of immunoreactivity for INI1 is a distinguishing (but not pathognomonic) feature of MRT. The disorder is associated with loss of the tumor suppressor SMARCB1/INI1/SNF5/BAF47 on chromosome 22q11 due to either heterozygous germline deletions or somatic mutations. 

##### Outcome

Rhabdoid tumors have the lowest survival rate (1 out of 24, 4%) of all malignancies at this age [7]. 

#### 2.2.4. Congenital Histiocytosis

##### Congenital Langerhans Cell Histiocytosis (LCH)

**Figure 7:**
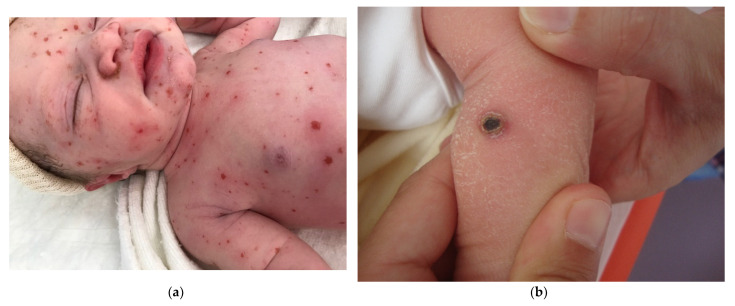

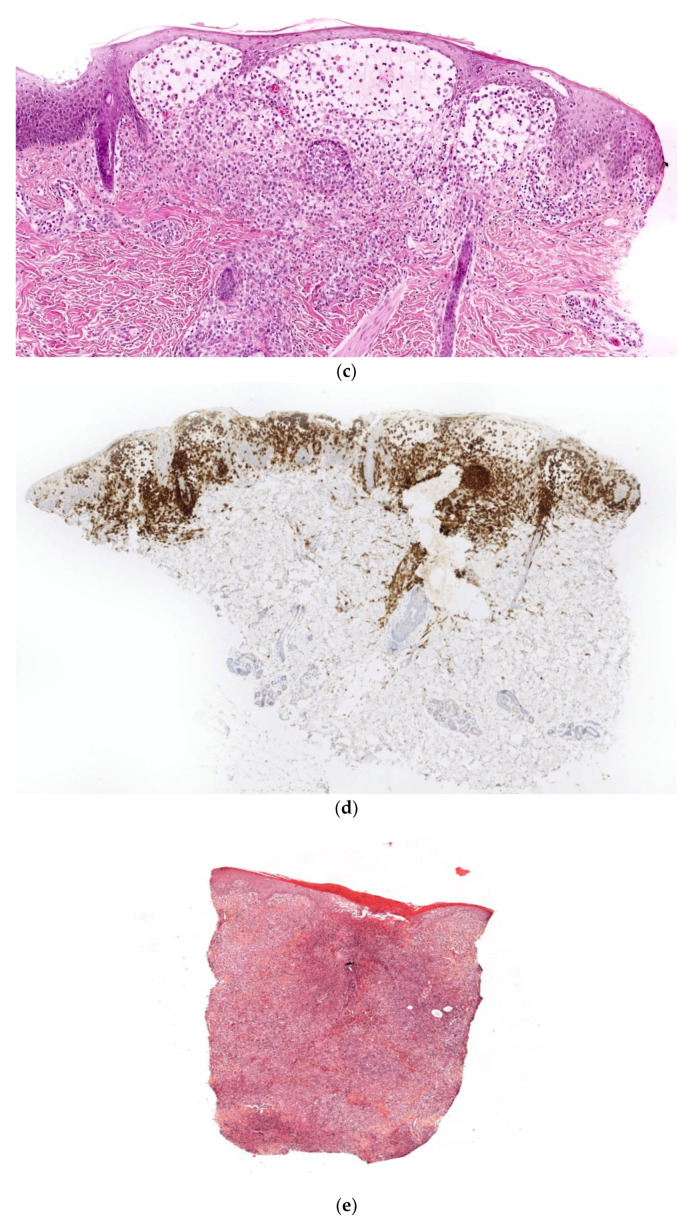
Langerhans cell histiocytosis (**a**) disseminated congenital lesions with erosive and purpuric lesions. (**b**) nodular and crusted lesion (Hashimoto-Pritzker). (**c**) 2.5× usual aspect of LCH with Langerhans cell infiltrate filling the papillary dermis. (**d**) 2.5× anti-CD1a. (**e**) 2.5× Nodular spontaneously regressive lesion. Dense infiltrate throughout the dermis with very few epidermotropism.

Congenital Langerhans cell histiocytosis (LCH) is rare and, in most cases, regresses spontaneously very quickly, within a few weeks of birth. This self-resolving form of histiocytosis was first described by Hashimoto and Pritzker in 1973. Typically, skin lesions manifest as widespread vesicles, pustules, or both. They are often erosive and purpuric and can be mistaken for neonatal herpes. Purplish, reddish-brown, crusted, papulonodular lesions with central necrosis and ulceration are observed in 25–30% of cases of neonatal LCH (Figure 7a,b). A few nodules may develop [11,12]. Furthermore, some babies with congenital LCH may develop a “blueberry muffin rash” [13]. 

##### Histology

A skin biopsy is always necessary to confirm the diagnosis of LCH. Cutaneous LCH is characterized by an infiltration of the papillary dermis by monomorphous, medium-sized histiocytes with a pink, non-xanthomized cytoplasm and a kidney-shaped, grooved, or folded nucleus. These cells are positive for CD1a and CD207 (Langerin); the latter is specific for Birbeck granules. These cells may be associated with eosinophils, lymphocytes, and/or red blood cells. Epidermotropism results in a spongiform pattern with focal parakeratosis. It is not possible to differentiate between regressing LCH and non-regressing LCH on the sole basis of the histological data. The nodular form of LCH shows a tumoral intradermal infiltration of large histiocytes and commonly ulcerated overlying epidermis. The cells comprise Langerhans cells (CD1a+, CD207+), indeterminate cells (CD1a+, CD207− (Figure 7c–e), and macrophages (CD163+, CD1a−, CD207−) [14]. Most cases harbor mutations in the MAP kinase pathway and especially in the *BRAF* gene.

##### Outcome

In most cases of LCH, the lesions observed at birth are not accompanied by systemic signs and tend to involute spontaneously within a few weeks or months. Since the original description, however, many cases of extracutaneous involvement, visceral involvement, and/or disease recurrence have been reported. It has not yet been possible to define clinical or histological factors that distinguish between the “self-healing” and progressive multisystem forms of neonatal LCH [15]. Accordingly, careful work-up and close follow-up are necessary in all cases.

##### Multiple Xanthogranuloma

**Figure 8:**
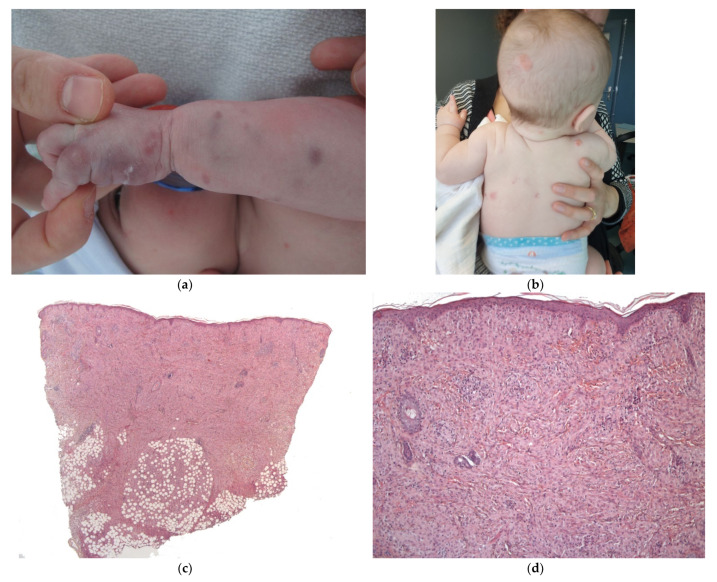

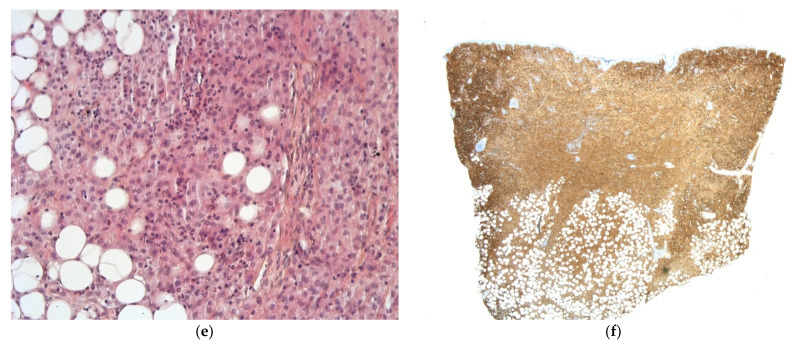
Juvenile xanthogranuloma. (**a**) Presenting as blueberry muffin rash (by courtesy E Puzenat); (**b**) multiple yellowish lesions. (**c**) 4× rather dense infiltrate in the dermis extending into the superficial subcutis. (**d**) 10× non xanthomized histiocytes with no giant cells (mononuclear-vacuolated early variant). (**e**) 25× Histiocytes are accompanied by inflammatory cells, in particular here, neutrophils. (**f**) Anti-Factor XIIIa positivity.

Juvenile xanthogranuloma (JXG) is a fairly common non-Langerhans cell histiocytosis. It most often affects infants and young children and is characterized by the dermal accumulation of variably xanthomized histiocytes. The skin lesions usually resolve, and most patients have an otherwise unremarkable clinical course. In total, 20% of cases of JXG are congenital, and multiple lesions are observed in 20% of congenital cases. Very rarely, the disorder also affects extracutaneous organs: mostly the eyes but also the liver, spleen, lungs, central nervous system, kidneys, retroperitoneum, or elsewhere in the body. JXG features small, smooth, and dome-shaped papules rather than large nodules. The color depends on the age of the lesions (i.e., the degree of xanthomization): first pink to red-brown, and then yellow (Figure 8a,b). Most lesions resolve spontaneously, albeit over months or even years.

##### Histology

Typically, the lesions demonstrate a well-demarcated, dense infiltrate of histiocytes within small lesions in the superficial dermis. Larger lesions extend into the deep dermis or subcutis. There is often loss of the rete ridges, and ulceration occurs in rare cases. Early lesions usually contain monomorphous histiocytes with abundant eosinophilic cytoplasm. In mature lesions, an accumulation of lipids in the histiocytes’ cytoplasm gives them a foamy “xanthomatous” appearance. Touton giant cells are common at this stage. Lymphocytes and eosinophils are also scattered throughout the infiltrate. In fact, all types and shapes of histiocyte can be present (Figure 8c–e).

These histiocytes may originate from a “dermal dendrocyte”; they are always strongly positive for CD163, sometimes positive for FXIIIa (Figure 8f), and always negative for CD1a and CD207. Although most patients with cutaneous JXG do not carry any mutations, mutations in the MAP kinase pathway have been described in some cases with multiple lesions and organ involvement [16].

##### Outcome

In patients with systemic disease, the clinical course can be catastrophic, and spontaneous remission is very rare [17]. The treatment of choice is a combination of vinblastin and dexamethasone. A BRAF inhibitor can be considered in patients with a mutation. Therefore, children with multiple lesions at birth—particularly those involving the eye—should always be screened for systemic involvement [18].

#### 2.2.5. Transplacentally Acquired Tumors

It has been estimated that 1 in every 1000 pregnancies will feature a malignancy, about the same frequency as in age-matched nonpregnant women. Melanoma is one of the least rare.

##### Transplacental Melanoma

Melanoma is the most common neoplasm with transplacental transmission to the fetus [19]. The estimated incidence of melanoma during pregnancy is 0.1 to 2.8 per 1000. Fetal metastases are rare; around 100 case reports have been published. Although spontaneous regression has very occasionally been reported, most neonates with clinical evidence of maternal metastases at birth have an exceedingly poor prognosis [20].

##### Histology

In some circumstances, the diagnosis of transplacental melanoma may be confirmed by biopsy. In most cases, the diagnosis is straightforward when pigmentary lesions are seen in a newborn born to a mother with confirmed metastatic melanoma.

##### Outcome

Death typically occurs before the age of 3 months. Placental involvement appears to be a key risk factor among patients with no clinical evidence of metastasis at birth [21].

##### Transplacental Choriocarcinoma

Infantile choriocarcinoma is a rare, highly malignant germ cell tumor that arises from the placenta. It is characterized by the secretion of human chorionic gonadotropin (hCG). Simultaneous intraplacental choriocarcinomas involving both the mother and infant are extremely rare, and cutaneous metastasis in infantile choriocarcinoma is even rarer [22,23]: fewer than 30 cases have been described in the literature. About half of these cases arise from a complete hydatidiform mole, a quarter arise after normal pregnancies, and a quarter appear after a spontaneous abortion or an ectopic pregnancy. The cutaneous manifestations of choriocarcinoma are metastatic nodules, subcutaneous masses, and multiple angiomatoid tumors.

##### Histology

**Figure 9:**
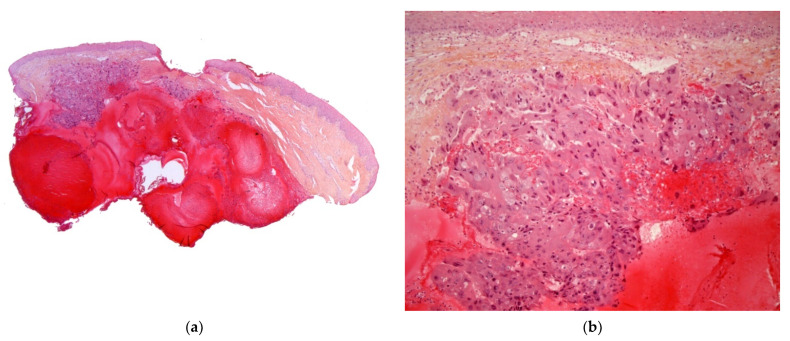
Metastatic choriocarcinoma. (**a**) 4× cellular and hemorrhagic areas in the reticular dermis arranged in lobules. (**b**) 25× very large typical cells of syncytiotrophoblast.

The two-zone tumor has a central core of mononuclear cytotrophoblasts and a peripheral rim of multinucleated syncytiotrophoblasts. Extensive hemorrhage and necrosis are frequent (Figure 9a,b). The trophoblasts show marked cytological atypia, and immunohistochemistry with antibodies against hCG or human placental lactogen confirms the diagnosis [23].

##### Outcome

The outcome is poor, with a survival rate below 25%. More recently, however, it has been shown that prompt diagnosis (from a biopsy of a metastatic skin nodule) and chemotherapy improve the prognosis significantly [23,24].

#### 2.2.6. Infantile Myofibromatosis

Infantile myofibromatosis (IM) is a benign mesenchymal tumor from the juvenile fibromatosis group and is commonly encountered by dermatologists. The name “infantile myofibromatosis” reflects the fact that the tumor is composed of myofibroblasts. Infantile myofibromatosis affects children during the first decade of life, and 60% are present at birth.

There are three defined clinical patterns: (i) solitary, self-limiting disease (also called myofibroma); (ii) multicentric disease (involving the skin, subcutaneous tissues, muscles and bones, with a typical benign course and spontaneous resolution); and (iii) a generalized form with visceral involvement and a poor prognosis [25]. The multifocal lesions may be quite numerous (from a few dozen up to 100 or more) and are present at birth. Girls are more commonly affected than boys.

**Figure 10:**
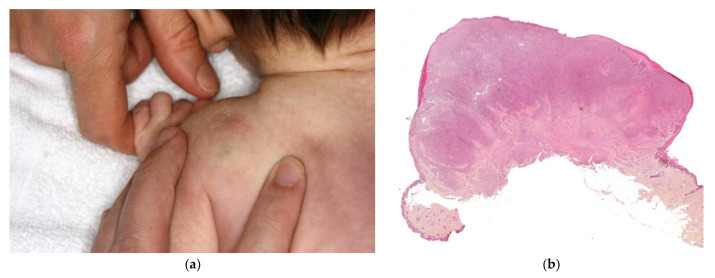

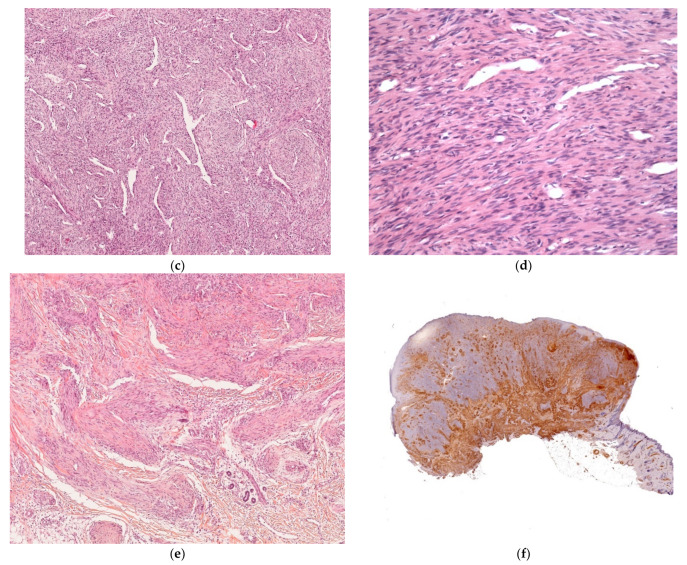
Infantile myofibromatosis. (**a**) This baby presented with multiple small blueish lesions. (**b**) 4× dermal rounded well-circumscribed lesion. (**c**) 10× antler-shaped vascular proliferation associated with spindle-shaped cells in the center of the lesion. (**d**) 25× spindle-shaped cells arranged in fascicles. (**e**) 10× round lobules made up of elongated cells with poorly seen cytoplasmic borders at the periphery of the lesion. (**f**) 4× anti-SMA.

There are many different clinical presentations, depending on the proportion of myofibroblasts and the degree of vascular proliferation. The lesions may present as (i) firm, flesh-colored papulonodules; (ii) crusted or angiomatous infiltrate plaques; (iii) pedunculated, infiltrated, calcified tumors; or (iv) ulcerated, necrotic, pseudo-sarcomatous tumors mimicking cutaneous metastases (neuroblastoma) and leukemia cutis (all causes of multiple nodules at birth) (Figure 10a). Hence, an appropriate biopsy is always necessary. 

##### Histology

The lesion is characterized by a two-zone pattern best seen at low power: a central hemangiopericytoma-like area surrounded by a leiomyoma-like area. The relative proportions of the two areas can vary. The periphery of the myofibroma is marked by the proliferation of short bundles of spindle cells with a myoid appearance (i.e., plump nuclei and abundant pale cytoplasm), which are frequently clustered into round aggregates. The tumor’s central region is often marked by a proliferation of rather small round-to-oval cells with little cytoplasm. Centrally located round-to-oval cells are often arranged around prominent “antler-shaped” vascular spaces (Figure 10b–e). This central region often develops areas of ischemic necrosis. Mitotic figures are not rare. Spindle cells are strongly positive for smooth muscle actin (Figure 10f) and calponin and, in some cases, desmin [25].

##### Outcome

Patients with multifocal disease still have an excellent prognosis; most experience spontaneous disease resolution over 12 to 18 months [25]. However, patients with generalized IM typically have severe systemic diseases. Visceral involvement can involve the gastrointestinal tract, lungs, heart, and upper respiratory tract [26]. Death occurs mainly within the first weeks of life and within 4 months at the latest.

Neonates with multiple lesions should undergo a full skin examination and imaging, to determine the extent of the disease. In patients with visceral involvement, systemic treatment may be necessary. The tumor typically responds slowly to conventional chemotherapy, and tumor reduction is usually not observed until after several weeks of treatment. Recently, somatic mutations in the gene coding for platelet-derived growth factor receptor beta polypeptide (PDGFR-β) have been identified in most sporadic multicentric forms of the disease. Hence, PDGFR inhibitors, like imatinib, have been considered for use in life-threatening cases. However, second-line treatment with a targeted therapy should only be considered in the context of life-threatening disease progression [27,28].

### 2.3. Non-Neoplastic Disorder: Subcutaneous Fat Necrosis of the Newborn 

This is a rare condition, characterized by multiple erythematous or skin-colored indurated subcutaneous nodules or plaques that appear during the neonatal period in a large hardened area on the back, buttocks, and limbs.

**Figure 11:**
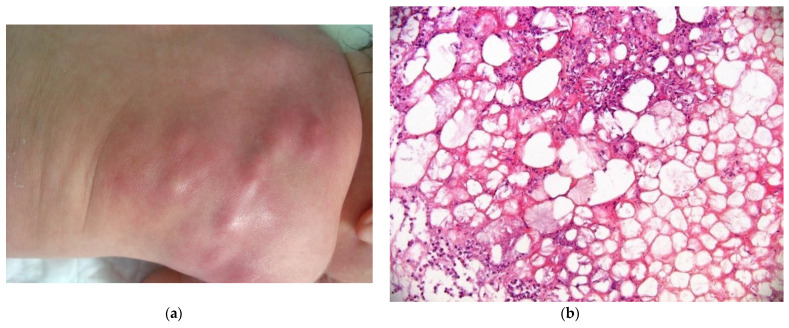
Subcutaneous fat necrosis of the newborn. (**a**) Multiple skin-colored nodular lesions at the upper back. (**b**) 10× lobular panniculitis with inflammatory cells and typical radially arranged needle-shaped cleft into the fatty tissue.

The lesions are primarily observed on the body areas with the most adipose tissue, such as the shoulders, buttocks, cheeks, and thighs (Figure 11a). The nodules frequently soften and become fluctuant, or occasionally liquefy. At birth, these nodules may mimic the lesions in leukemia cutis or neuroblastoma metastases; a biopsy may then be necessary. 

#### 2.3.1. Histology

The biopsy of an early lesion always has a characteristic appearance: a dense, inflammatory, lobular infiltrate in the subcutis is composed of lymphocytes, histiocytes, eosinophils (occasionally), and multinucleated giant cells with fat necrosis. Radially arranged needle-shaped clefts, corresponding to crystallized fatty acids that were dissolved during slide processing, are seen within the adipocytes and giant cells [29] (Figure 11b).

#### 2.3.2. Outcome

The lesions resolve spontaneously within a few weeks or months.

## 3. Multifocal Vascular Skin Lesions

Multifocal vascular lesions at birth may correspond to different entities of different prognosis and therapeutic management (Table 2).

### 3.1. Multifocal Infantile Hemangioma

**Figure 12:**
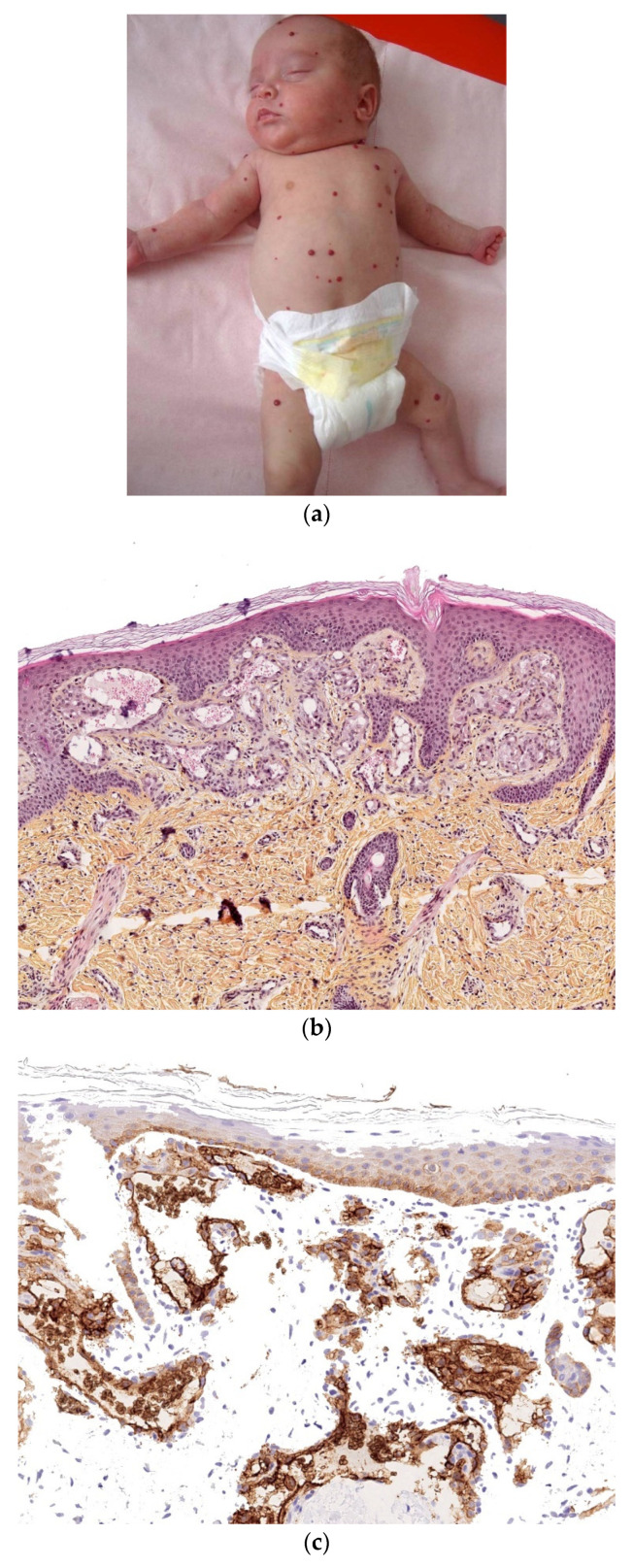
Multifocal infantile hemangioma. (**a**) Multiple small red lesions disseminated over the body. (**b**) 10× Well-differentiated small vessels in the superficial dermis. (**c**) Anti-Glut1 immunostaining showing positivity of all the endothelia.

Multifocal infantile hemangiomas develop shortly after birth [30], ranging from several to hundreds. These dome-shaped, bright red papules or nodules vary in size from a few millimeters to centimeters but tend to be small, extraordinarily numerous, spread all over the body, and superficial (Figure 12a).

#### 3.1.1. Histology 

The histological features of multifocal infantile hemangioma are identical to those of solitary infantile hemangioma, except that capillaries are well differentiated from the outset and are arranged in lobules within the dermis, usually the superficial dermis (Figure 12b). The lesions are always positive for Glut1 (endothelial staining) (Figure 12c).

#### 3.1.2. Outcome

The lesions regress spontaneously as early as the first year of life and often have involuted completely by 2 years of age. The presence of more than five cutaneous infantile hemangiomas is a potential marker for hepatic involvement, and an abdominal ultrasound assessment is indicated. Depending on their number, density, and severity, the hepatic lesions are usually asymptomatic but can cause serious complications (such as heart failure). 

Patients with widespread hepatic involvement can be treated with propranolol [31].

### 3.2. Multifocal Lymphangiomatosis with Thrombocytopenia

**Figure 13:**
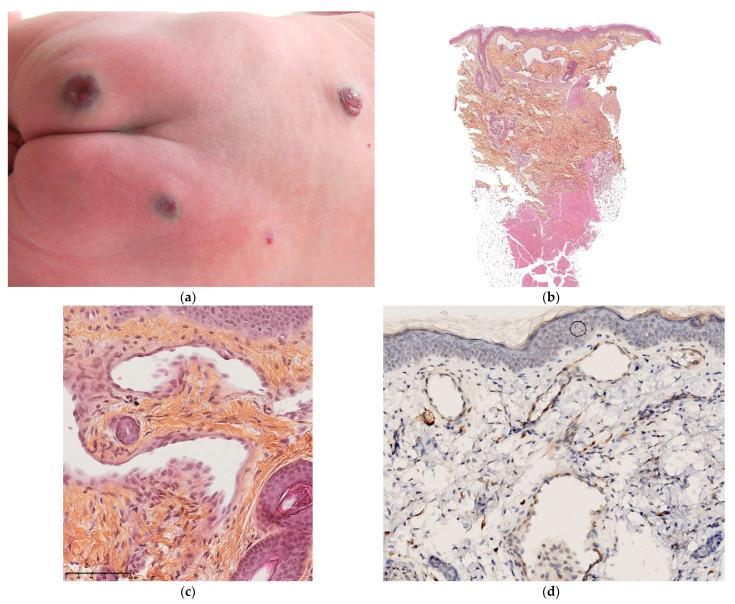
Multifocal lymphangiomatosis with thrombocytopenia. (**a**) Red-brown papules and plaques (by courtesy C Droitcourt). (**b**) 2.5× Thin-walled vascular channels dissecting the superficial and mid-dermis. (**c**) 4× These vessels are lined by endothelial cells with a plump, sometimes hobnail, nucleus. (**d**) These cells are positive for Lyve 1.

Multifocal lymphangiomatosis with thrombocytopenia (MLT) is a vascular disorder affecting several organs. It is associated with thrombocytopenia, the severity of which may vary. Classically, both the skin and the gastrointestinal tract are involved [32]. The skin lesions are present at birth and appear as multifocal, discrete, and red-brown to burgundy papules, plaques, and nodules, ranging from a few millimeters to several centimeters in size (Figure 13a) [33]. The number of lesions ranges from a few to hundreds. The trunk and limbs are more commonly involved. Gastrointestinal tract lesions present with hematemesis and/or melaena, usually early in infancy. Endoscopy reveals a few or many small vascular lesions on the gastrointestinal mucosa. Additional lesion sites include the lung, bone, liver, brain, synovium, and muscle. Thrombocytopenia is most commonly present in the first month of life. It is usually sustained, with a mean platelet count of 50,000–100,000/ mL, and results in gastrointestinal tract bleeding. Fibrinogen levels are normal to low, and D-dimer levels may be elevated.

It is important to distinguish MLT from other multifocal congenital vascular diseases that affect the skin and viscera, such as multifocal venous malformations (e.g., blue rubber bleb nevus syndrome) and multiple congenital pyogenic granulomas. Hence, a biopsy is always required.

#### 3.2.1. Histology

A histologic assessment of MLT will reveal dilated, thin-walled vascular channels and variable endothelial hyperplasia in the dermis and subcutis. Most lesions display intraluminal papillary projections [32,33] (Figure 13b,c). The endothelial nuclei are prominent and sometimes hobnailed. MLT is often positive for the lymphatic marker Lyve-1 but may be negative for podoplanin (using the D2-40 antibody) (Figure 13d).

#### 3.2.2. Outcome

MRI and computed tomography are used to document the extent of the disease in patients with MLT. The lesions may develop progressively throughout the body or may stabilize or regress over time [32,33]. Treatment strategies are not well established because of phenotypic variations and the rarity of this disease. Blood and platelets can be administered. Sirolimus appears to be promising. In one study, the vascular lesions responded fully or partly to treatment, leading to clinical improvement in a small number of patients [34].

### 3.3. Multiple Neonatal Pyogenic Granulomas

Congenital disseminated pyogenic granuloma is a distinctive, multisystemic aggressive disorder that primarily affects the skin, brain, visceral organs, and musculoskeletal system [35].

**Figure 14:**
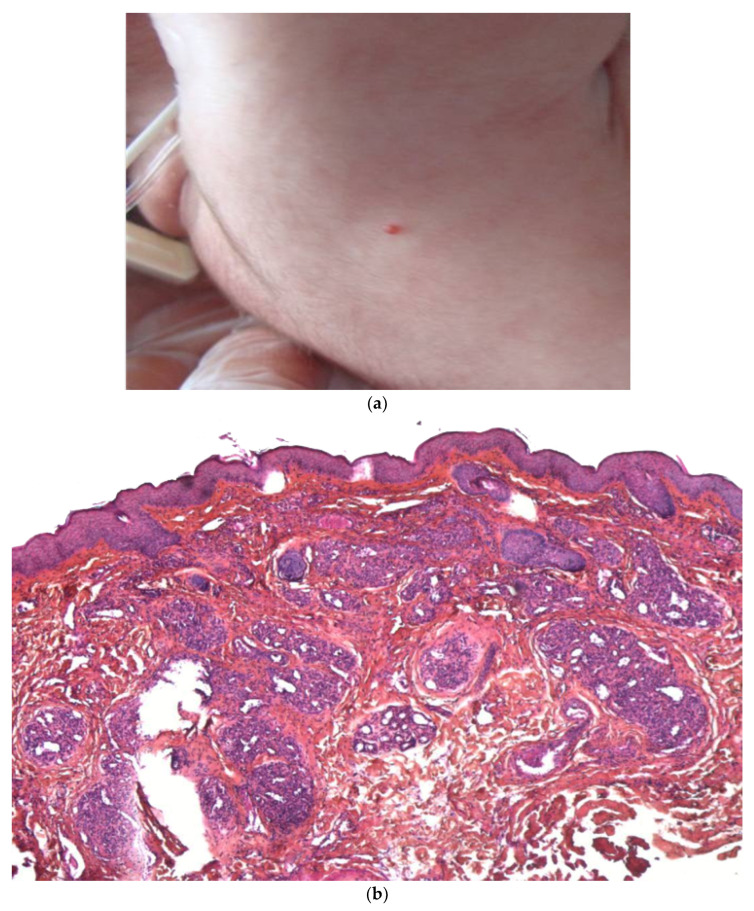

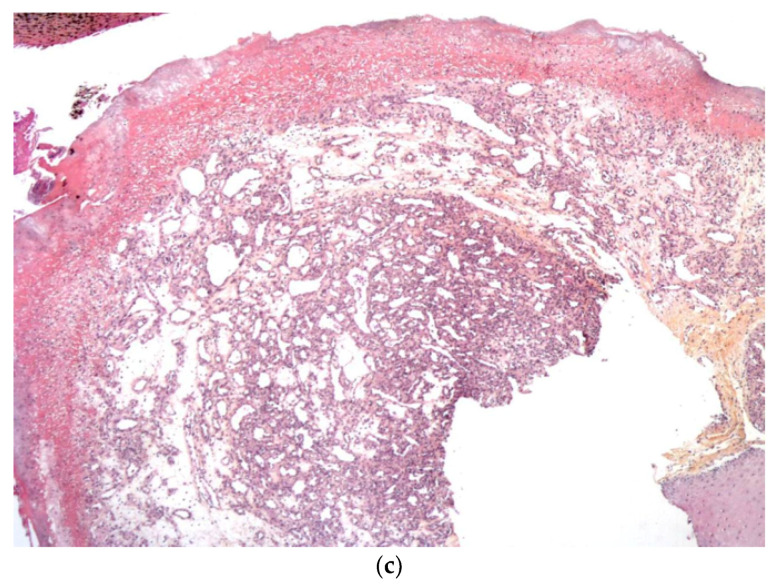
Neonatal pyogenic granulomas. (**a**) Small red typical pyogenic granuloma. (**b**) 4× lobulated vascular lesion in the dermis. The overlying epidermis is normal whereas in (**c**), it is ulcerated and the lesion contains inflammatory cells.

The granulomas commonly affect the skin and the epithelia mucosa. Multiple congenital pyogenic granuloma is very rare but must be accounted for as a diagnosis because extracutaneous organs (such as the brain and liver) may be affected. The lesion is typically a bright red, friable papule that grows quickly and is prone to bleeding. A loosely adherent hemorrhagic crust may be seen. Lesions may be pedunculated or sessile (Figure 14a) and most are less than 1 cm in diameter [36]. Differentiation of this entity from other multiple cutaneous vascular lesions is critical because of possible cerebral hemorrhagic involvement. A tissue biopsy is essential for a definitive diagnosis.

#### 3.3.1. Histology

A histologic assessment shows a well-circumscribed lobular proliferation of capillaries. The lobules are separated by bands of fibrous stroma. An epidermal collarette, ulceration, and inflammation are additional features (Figure 14b,c). The endothelial cells are always negative for Glut1 and podoplanin.

#### 3.3.2. Outcome

Spontaneous regression is usually observed.

### 3.4. Multiple Tufted Angiomas/Kaposiform Hemangioendotheliomatosis

Multiple tufted angiomas (TAs) and kaposiform hemangioendotheliomatosis (KHE) are related vascular tumors that present in infancy and childhood. They share histological features (including the proliferation of blood vessels and lymphatic vessels) and are now considered to different clinical expressions along the spectrum of the same vascular anomaly. Multifocal presentation is very rare. Clinically, multiple reddish-blue skin nodules are observed. Histopathologic assessment is mandatory because TA/KHE is a differential diagnosis for other multifocal vascular disorders [37,38].

#### Histology

A histological assessment of TA shows nodules of densely packed endothelial cells that are scattered throughout the dermis in a “cannonball” pattern. The lobules are surrounded by crescent-shaped vascular spaces. The endothelial cells are only partially canalized, and the vessels have a slit-like appearance reminiscent of Kaposi sarcoma. KHE has more infiltrating sheets and lobules of tightly packed, spindle-shaped endothelial cells associated with microthrombi, hemorrhagic areas, lymphatic-like vascular spaces, and hemosiderin deposits.

**Figure 15:**
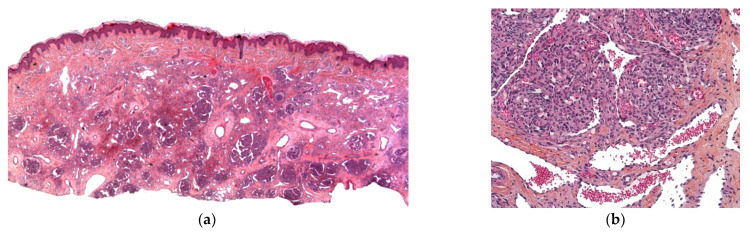

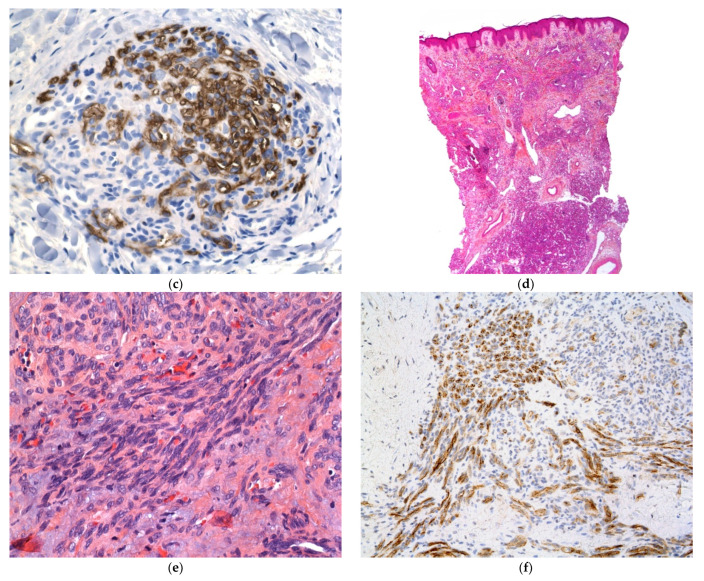
Tufted angioma and kaposiform hemangioendotheliomatosis. (**a**) 25× numerous very small and well-limited lobules throughout the dermis. (**b**) 25× packed small vessels and crescent-shaped vascular channel surrounding it. (**c**) Anti-podoplanin staining the lobules partially. (**d**) Poorly limited and infiltrative vascular tumor in the dermis extending into the subcutis. (**e**) Spindle-shaped cells with red blood cells in between. (**f**) Partial staining of the vessels with anti-podoplanin.

TA and KHE have a similar immunophenotype, with a positive staining for CD31, CD34, and, in all cases, the lymphatic marker podoplanin in the lobules and some extralobular vessels (Figure 15a–f).

### 3.5. Venous Malformations

TA and KHE are congenital disorders that can present with multiple deep cutaneous lesions. Multiple venous malformations can be associated with blue rubber bleb nevus syndrome, cutaneomucosal venous malformation, or glomuvenous malformation. The venous malformations are typically bluish, soft, and compressible.

#### 3.5.1. Glomuvenous Malformation

**Figure 16:**
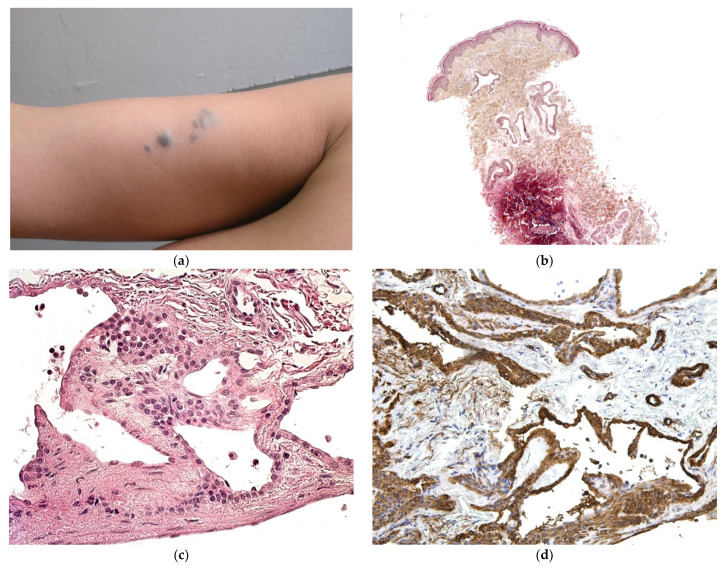
Glomuvenous malformation. (**a**) Bluish to violet small nodules in the arm. (**b**) 10× multiples veinous channels in the dermis. (**c**) 25× these vessels are surrounded by round glomus cells. (**d**) These cells are highlighted by anti-SMA.

This disorder is inherited in an autosomal dominant fashion. It only affects the skin and never the viscera. Congenital forms are very rare and are difficult to diagnose clinically because the regional or multifocal lesions are pink and not blue [39]. In most cases, new lesions appear after birth (Figure 16a).

##### Histology

A biopsy is always required for a firm diagnosis. It will typically show clusters of glomus cells situated around dilated vascular structures (Figure 16b–d). Immunohistochemically, the clusters are positive for smooth muscle actin.

#### 3.5.2. Blue Rubber Bleb Naevus Syndrome (Bean’s Syndrome)

**Figure 17:**
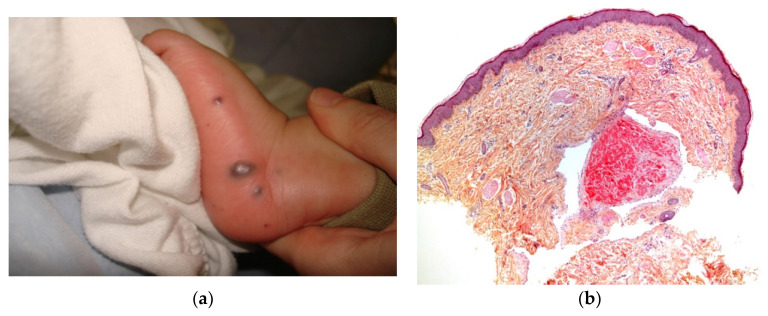
A blue rubber bleb nevus syndrome. (**a**) Blue nodules on the foot. (**b**) 4× dilated venous vessels, some of them containing thrombi.

Blue rubber bleb nevus syndrome is a rare vascular syndrome characterized by the continuous eruption of vascular nodules in the skin, mucous membranes, and solid organs due to somatic activating mutations in the gene coding for the angiopoietin receptor TEK (tunica interna endothelial cell kinase). The gastrointestinal tract, muscles, joints, central nervous system, eyes, parotid gland, spine, kidneys, and lungs may occasionally be affected. Skin lesions consist of soft blue nodules that occasionally aggregate into large masses. The small, colorless, dome-shaped, nipple-like lesions (“rubber blebs”) (Figure 17a) are often associated with a large “dominant” venous malformation.

##### Histology

A histological assessment shows multiple dilated venous structures in the dermis, with no glomus cells (Figure 17b). 

##### Outcome

Blue rubber bleb nevus syndrome may be complicated by acute, life-threatening hemorrhage and localized intravascular coagulation. Sirolimus may be considered as a first-line treatment, depending on the severity of the disease [40].

## 4. Cutaneous Mastocytosis

**Figure 18:**
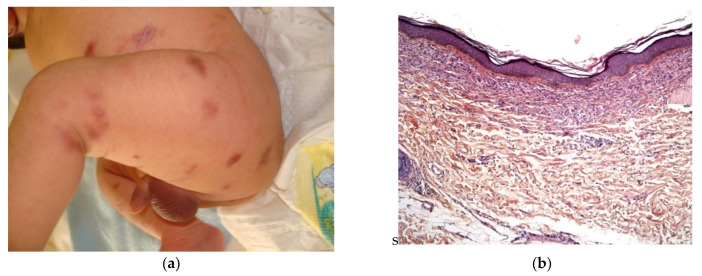

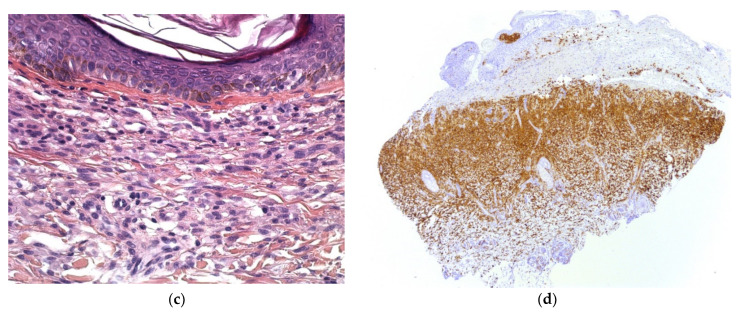
Cutaneous mastocytosis. (**a**) Multiple maculopapular lesions present at birth. (**b**) Rather dense band-like infiltrations at the upper dermis. (**c**) 40× large mast cells containing granules in their cytoplasm. (**d**) anti-CD117 stains mast cells in bullous mastocytosis.

Around 30% of cases of pediatric cutaneous mastocytosis are present at birth. The lesions can be isolated and nodular and so are referred to as mastocytomas. However, other forms, such as maculopapular cutaneous mastocytosis, formerly referred to as urticaria pigmentosa, or diffuse cutaneous mastocytosis, can occur [41]. Maculopapular cutaneous mastocytosis manifests itself as multiple small nodules; this condition is very rare and may be difficult to distinguish from multiple xanthogranulomas, due to its yellowish hue. Darier sign is not always present. A biopsy is often necessary (Figure 18a).

### 4.1. Histology

Biopsies from neonatal cases of mastocytosis are usually easy to interpret. They show a large number of mast cells in the superficial dermis and mid-dermis that sometimes extend towards the subcutis (mostly surrounding the vessels) (Figure 18b,c). The cells stain positive for CD117 (c-kit) (Figure 18d).

### 4.2. Outcome

The outcome in neonatal cases is usually favorable, and the lesions regress before puberty [41]. 

## 5. Conclusions

Neonatal conditions with multiple cutaneous nodules often have a poor prognosis and thus require prompt diagnosis. A skin biopsy should always be part of the initial examination because the lesion’s histology will determine which further diagnostic investigations are required. Given that some congenital skin conditions (neoplastic or not) resolve spontaneously, prompt recognition and diagnosis are required.

## Figures and Tables

**Table 2:** Multifocal neonatal vascular skin lesions.

Multifocal infantile hemangioma
Multifocal lymphangiomatosis with thrombocytopenia
Multiple neonatal pyogenic granuloma
Multiple tufted angioma/kaposiform hemangioendothelioma
Venous malformations:
glomuvenous malformation
blue rubber bleb naevus syndrome (Bean’s syndrome)
